# Put Me in the Sky^1^

**DOI:** 10.3201/eid1511.000000

**Published:** 2009-11

**Authors:** Polyxeni Potter

**Affiliations:** Centers for Disease Control and Prevention, Atlanta, Georgia, USA

**Keywords:** Art science connection, emerging infectious diseases, art and medicine, Romare Bearden, Circe Turns a Companion of Odysseus into Swine, global wildlife trade, Odysseus series, about the cover

**Figure:**
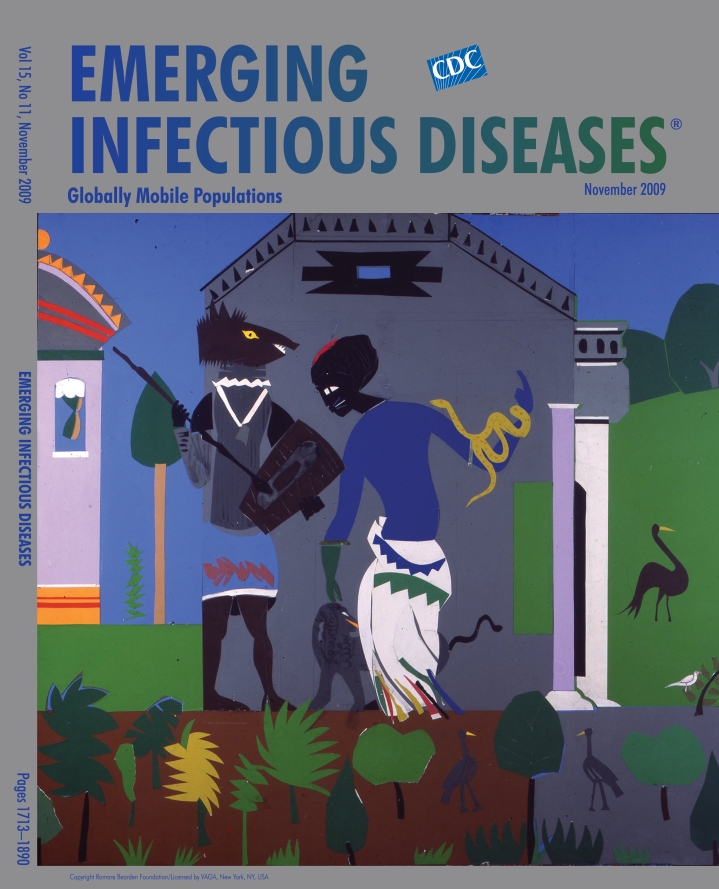
**Romare Bearden (1911–1988) Circe Turns a Companion of Odysseus into Swine (1977)** Collage of papers with paint and graphite on fiberboard (81.3 cm × 111.8 cm) Copyright Romare Bearden Foundation/Licensed by VAGA, New York, NY, USA

“I thought that I was going to be a doctor, and I majored in science and later in mathematics. But when I got out of college, I decided to study art,” divulged Romare Bearden in a 1968 oral history interview. In New York, “I went to study with George Grosz at the Art Students League.” The German-born artist guided Bearden’s understanding of draftsmanship and classical technique, introducing him to Ingres, Hogarth, Holbein, and Dürer and urging him to “really observe.”

“I was born in Charlotte, North Carolina …. I grew up mostly in New York and some time in Pittsburgh, where I would go to see my grandmother,” Bearden said about his peripatetic childhood. But he added on another occasion, “I never left Charlotte except physically.” His family, part of the Great Migration north to escape racial segregation, became prominent in their new community, sharing in the intellectual, artistic, and political mainstream of the 1920s and ’30s cultural movement, Harlem Renaissance.

“When I finished studying with Grosz, I drew at home and I got a job as a political cartoonist.” But painting was what he wanted to do, so he got a studio in New York, same building as Jacob Lawrence and other artists, writers, and musicians, who became his friends. “I began at that time to do my Southern themes, the people that I’d seen as a young boy when I’d sometimes visit North Carolina ….” This promising career was interrupted by World War II, when he joined the army.

“After the war, I began to arrive at some kind of personal identification.” Like many in his circle, he traveled to France, “Paris was just like a thing of dreams to me.” But despite his fascination with the city and the people he met, among them Georges Braque and Constantin Brâncuşi, he did not paint at all there. Back in New York, he gradually overcame his desire to return to Paris and started to paint again, experimenting with color “not as decoration” but as “form, as space,” with “flat painting, shallow space, Byzantine stylization, and African design.”

“I think the artist has to be something like a whale, swimming with his mouth wide open, absorbing everything until he has what he really needs.” Bearden dabbled in many techniques and media, enriching them with his understanding of literature, music, and philosophy. He wrote successfully and had to be dissuaded from pursuing music so that he would remain focused on art. He grew up listening to Duke Ellington’s orchestra and Ella Fitzgerald’s singing. For 16 years, his studio was above the Apollo Theater, a Harlem landmark. “I paint out of the tradition of the blues,” he wrote. “The more I played around with visual notions as if I were improvising like a jazz musician, the more I realized what I wanted to do as a painter, and how I wanted to do it.”

In the 1960s, he moved away from painting toward more structured and varied compositions that better accommodated his conflicting interests in representation and abstraction. His richly textured collages, which captured every aspect of that turbulent era, contained, among other bits and pieces, painted images from other art works or photostatic enlargements made from photographs of these works. These enlargements, or Projections as they were called, featured in spectacularly complex works that synthesized old and new concepts: Manet’s Olympia in the Civil Rights Era, Matisse in rural North Carolina, Poseidon in New York. “I try to show that when some things are taken out of the usual context and put in the new, they are given an entirely new character.”

During the latter part of his life, Bearden often visited the Caribbean island of St. Martin. There he explored in depth a favored theme: human migration and the search for home. Near the water, for him a source of energy, he conceived his Odysseus series, 20 large collages and other smaller works inspired by Homer’s epic recounting the hero’s 10-year quest for Ithaca on the way back from the Trojan War.

“How you have gotten it! It’s all here, all right,” wrote Nobel Laureate Derek Walcott in his poem, “To Romare Bearden,” praising the artist’s genius and his ability to project humanity in visual, plastic terms. Intrigued by the universality of Homeric themes and their applicability to pressing issues of all time, the artist painted them with vigor and wit. “I am trying to explore, in terms of the particulars of the life I know best, those things common to all cultures.”

In Circe Turns a Companion of Odysseus into Swine, on this month’s cover, Bearden revisits this episode in the Odyssey with the clarity of a draftsman and the elusiveness of a storyteller. His view is theatrical, the luscious set strewn with clues. At Circe’s palatial edifice in the forest glade, the sky is brilliant against a slice of pink and a window curtain blowing in the breeze. At center stage, the goddess approaches an intruder. Face whiskered, arm snaked, she is poised to strike, has already, man into swine. Lawn and forest are littered with birds. In this flat sea of static figures, Bearden navigates beauty, magic, horror.

Circe “of the braided tresses” and her island, a major stop on Odysseus’ crowded itinerary, have had many interpretations. This granddaughter of the sun, goddess, enchantress who transforms men into animals and birds, cajoles the crew into a prolonged unscheduled stop and then, reluctantly, guides the hero’s exit to the sea. “Don’t evade, don’t pretend you won’t leave after all: you leave in the story and the story is ruthless,” laments Canadian author Margaret Atwood’s Circe, the one left behind.

“Needle in air, I stopped what I was making,” the goddess reports in Eudora Welty’s version of the episode, alluding to Odysseus’ arrival, her hospitality, the loom, the kitchen, the garden, the beauty of the island. This archetypal female character is entangled in the essence of Homer’s epic: the yearning for freedom and immortality that puts humans on paths riddled with danger and death. “Put me in the sky,” Welty’s Circe asserts, following the story line, frustrated with the turn of events. She wants to escape, become a distant constellation. I could have had “a ship too,” she professes, “if I were not tied to my island, as Cassiopeia must be to sticks and stars of her chair.”

Odysseys trace the paths of human migration: escape from war or segregation, poverty and famine; exploration of the earth and beyond; restlessness and search for meaning; hearth and harbor―the circuitous return to Ithaca, what a school of lessons! Magic and horror dominate. Dangers lurk en route. In our “Projection after Bearden,” this issue alone, they rival any epic’s: pandemic (H1N1) 2009, imported poliomyelitis, hepatitis A, Buruli ulcer, Mayaro fever, melioidosis, spreading multidrug-resistant TB, gastroenteritis at tourist resorts, dengue fever, malaria in refugees, low immunity to measles and rubella among guest workers, hepatitis E caused by a virus thought to come from pigs in the first outbreak reported on a cruise ship.

Not the least of plagues emerging around the globe are zoonotic infections due, not to travel by the animals so much as to their indiscriminate trade by humans. But even this twist is not beyond the scope of ancient epics. “It’s the animals I’m afraid of,” warned Atwood’s Circe, turning Odysseus’ story on its ear, “... they may transform themselves back into men.”
